# The Global Properties of Objects Play the Main Role in Facilitating Multiple Object Tracking Performance

**DOI:** 10.3389/fpsyg.2019.00924

**Published:** 2019-04-26

**Authors:** Liuqing Wei, Xuemin Zhang, Zhen Li, Bin Hu, Xiaowei Li

**Affiliations:** ^1^Department of Psychology, Faculty of Education, Hubei University, Wuhan, China; ^2^Beijing Key Laboratory of Applied Experimental Psychology, National Demonstration Center for Experimental Psychology Education, Faculty of Psychology, Beijing Normal University, Beijing, China; ^3^State Key Laboratory of Cognitive Neuroscience and Learning, IDG/McGovern Institute for Brain Research, Beijing Normal University, Beijing, China; ^4^Center for Collaboration and Innovation in Brain and Learning Sciences, Beijing Normal University, Beijing, China; ^5^eMetric, LLC., San Antonio, TX, United States; ^6^Gansu Provincial Key Laboratory of Wearable Computing, School of Information Science and Engineering, Lanzhou University, Lanzhou, China; ^7^CAS Center for Excellence in Brain Science and Intelligence Technology, Shanghai Institutes for Biological Sciences, Chinese Academy of Sciences, Shanghai, China; ^8^Beijing Institute for Brain Disorders, Capital Medical University, Beijing, China

**Keywords:** multiple object tracking, compound stimuli, global property, local property, global superiority

## Abstract

Previous research has revealed the uniqueness-facilitation effect in the multiple object tracking (MOT) task: simple distinct identities and surface features of moving targets could facilitate attentional tracking. By adapting compound stimuli, the present study investigated whether the global or local properties played the main role in the uniqueness-facilitation effect in the MOT task. The uniqueness of local properties, of global properties or of both local and global properties were considered. Observers’ tracking performance in alternative conditions were compared with that in the homogeneous condition wherein all stimuli have identical local and global properties. Results from two experiments suggest that the global properties played the key role in facilitating tracking. The distinctiveness of local properties can also facilitate tracking with global properties being homogeneous. However, when the stimuli’s global properties are distinct from each other—whether the local properties being unique or not—observers’ tracking performance can achieve the same level as that in the unitary-uniqueness condition wherein the moving objects were distinct unitary letters. These results revealed a global superiority effect in the MOT task. Finally, the facilitation effects of the global and local properties might depend on the stimulus sparsity. When the compound stimuli had fewer local elements, the uniqueness facilitation effect on tracking decreased.

## Introduction

Previous studies have shown that Human’s visual system can simultaneously keep track of several discrete moving objects (Pylyshyn and Storm, 1988; Scholl and Pylyshyn, 1999; Scholl et al., 2001; vanMarle and Scholl, 2003; Horowitz et al., 2007). They provided evidence that in the multiple object tracking (MOT) task, observers can localize a number of independently and unpredictably moving identical targets in a field of identical distractors. Researchers also pointed out that the attentive tracking in the MOT task was object-based, that is, attention must be allocated to the objects rather than the “substance” that extended and contracted during motion (vanMarle and Scholl, 2003) or the arbitrary collection of features (Scholl et al., 2001). Using the dual-task paradigm of the MOT and the probe dot detection task, Pylyshyn (2006) found that the probe on the targets was detected better than that in the space of near-targets, and the probe on the distractors was detected worse than that in the space of near-distractors, suggesting that both the attentional enhancement of targets and the attentional inhibition of distractors during tracking are also highly localized object-based.

In the earlier MOT task, the targets and distractors are all identical. That is, observers must monitor the trajectories of the moving targets constantly to localize them. However, recent research has considered whether objects were content-addressable during attentive tracking when objects differentiated from each other with unique identities or surface features, and whether objects’ identities or surface features had influence on attentive tracking. Pylyshyn (2004) in his study indicated that observers had difficulty identifying the targets even if they could successfully track their locations. However, the major reason of this result might be that the identities of targets were presented only briefly at the targets designation stage, but not in the tracking phase, making the maintenance of targets’ identities decayed during tracking. On the contrary, later studies in which objects’ identities or surface features were presented throughout the tracking phase showed that identities did exert an influence on the object-based attentive tracking, even when the identities of objects were not relevant to the task demand (Horowitz et al., 2007; Makovski and Jiang, 2009a,b; Howe and Holcombe, 2012). For instance, Horowitz et al. (2007) used cartoon animals as tracking stimuli and found that observers’ tracking performance improved when the objects were changed from identical objects or animals shared by the distractors to unique animals. That is, observers had an access to objects’ identities during tracking and utilized their uniqueness to aid tracking. Makovski and Jiang (2009a,b) showed that observers’ tracking performance was enhanced when the objects were of unique colors or 1-digit numbers, relative to that when the objects consisted of the same features or when the targets’ features paired with the distractors. And they revealed the cause to be the visual working memory mechanism operating parallel to the attentive tracking. Specifically, observers can store the targets’ unique identities into the visual working memory, once one or more targets were lost during tracking, they could search for and find them back based on the initially stored identities. However, later studies provided evidence that not all uniqueness of identities could facilitate tracking. When the objects carried on complex identities, such as numbers of three- or four-digit length, complex Chinese characters and human faces, the uniqueness of identities would impair tracking (Ren et al., 2009; Liu et al., 2012). One possible explanation is that the processing of complex identities consumed extra resources and had a small capacity in visual working memory (Liu et al., 2012).

Recent studies demonstrated the feature-based grouping effect in the MOT task (Erlikhman et al., 2013; Wang et al., 2016). They found that when all targets shared one feature and all distractors shared another—for instance, all targets were red circles and all distractors were green circles—observers’ tracking performance could be enhanced. Furthermore, when the targets belonged to one semantic category (e.g., animals) and the distractors belonged to another (e.g., furniture), even when they all had unique identities, this categorical distinction between the targets and distractors could also aid location tracking and suggested a semantic category-based grouping mechanism (Wei et al., 2016, 2017, 2018).

In light of the above findings, objects’ simple unique identities or features can facilitate location tracking in the MOT task, even when their identities or features are irrelevant to the task demand. That is, the processing of identities or features is involuntary and to some extent at an implicit level (Horowitz et al., 2007; Cohen et al., 2011).

Previous research mainly manipulated the uniqueness of objects’ simple surface features (e.g., color) or identities (e.g., unique animals) to test their effects on attentional tracking. But it is unknown which aspect of the visual objects, the local or global properties, played the main role in the uniqueness-facilitation effect. According to the global precedence hypothesis in the framework of Gestalt psychology (Navon, 1977; Wagemans et al., 2012b), a visual object can be viewed as represented by a hierarchical network with two levels, the global properties corresponds to the top level of the hierarchy while the local properties corresponds to the bottom level of the hierarchy. The present study did not concentrate on the order of processing of a visual object, but mainly explored the following issues: Did a “global advantage” also exist in the MOT task? That is, could the uniqueness of global properties produce much stronger tracking facilitation effect than that of local properties? Could the uniqueness of local properties facilitate object-based tracking? To test these issues, two experiments were conducted: Experiment 1 used the Navon-type compound stimuli (Navon, 1977) and tested whether the global or local properties played a major role in facilitating tracking. Experiment 2 used the sparse compound stimuli to further explore the same questions.

There are the two reasons why we choose the compound stimuli as tracking stimuli in the MOT task. First, the compound stimuli can be equated across stimuli in terms of complexity, familiarity, codability and identifiability (Navon, 2003; Wagemans et al., 2012b). Second, the real-world stimuli’s wholistic properties and component properties vary on a number of factors, making it hard to manipulate their homogeneity or heterogeneity. On the contrary, the compound stimuli have two distinct levels of structure—global configuration and local elements, which are relative independent (Kimchi, 1992; Navon, 2003), thus they provide a convenient way to segregate the global and local properties and test their respective effects on tracking.

## Experiment 1

Experiment 1 aimed to test two questions: (a) Could the uniqueness of objects’ local properties facilitate tracking? (b) Did global properties or local properties play the main role in facilitating tracking? The compound stimuli were used as tracking stimuli. The uniqueness of local properties, of global properties or of both of them varied across four conditions.

### Methods

#### Participants

The sample sizes in the present study were decided mainly by reference to previous research, in which there are usually 10–20 participants for the MOT task. Variability in the sample size of each experiment reflects the variability of available participants in the participant pool. A prior power calculation was also conducted investigating the required sample sizes (Faul et al., 2007). Since the MOT task was adapted from our previous research (Wei et al., 2018), which has found effect sizes (partial $\eta_{p}^{2}$) in repeated measures ANOVA of 0.50 or larger. To obtain a power of 90% at $\eta_{p}^{2}$ = 0.50, a sample of 5 was required.

According to standard practice, 19 students (10 female, 9 male), 18–26 years old (mean age = 22.00, *SD* = 2.11) with normal or corrected-to-normal vision, participated in the experiment. Two participants’ data was excluded from analyzing for whose tracking accuracies were under 50%. All observers provided informed written consent. The study was approved by the institutional review board (ethics committee) of the Faculty of Psychology at Beijing Normal University. All observers received payment for their time.

#### Equipment and Stimuli

##### Equipment

The experimental task was controlled by software written in Microsoft Visual Basic.NET (version 2013). The stimuli were presented on a Founder 17-in. CRT monitor with a resolution of 1024 pixels × 768 pixels and a refreshing rate of 85 Hz. Observers responded by pressing buttons on a keyboard and a mouse.

##### Stimuli

Moving stimuli were presented in a white, rectangle display that subtended 1024 pixels × 768 pixels (40.96° × 30.72°). A gray cross subtended 30 pixels × 30 pixels (1.2° × 1.2°) was presented in the center of the display as the fixation. The compound stimuli were lager letters (the global level) composed of small identical letters (the local level). The eight lager letters were A, E, F, H, L, S, T, and V, which consisted of the same small letters of A, E, F, H, L, S, T, and V. Thus, there were totally 64 compound stimuli. The unitary stimuli were eight capital letters. The larger letters measured 90 pixels (3.6°) vertically, and the smaller letters measured about 11 pixels (0.45°) vertically.

The stimuli moved at a constant speed of 17°/s. A repulsion technique was adopted in order to keep the stimuli from colliding. The stimuli bounced off each other when their center-to-center distance was less than 90 pixels. They also avoided the edge of the display area.

#### Design

In the current study, we mainly manipulated the uniqueness of local properties or global properties between the targets and distractors through five conditions: homogeneous, local uniqueness, global uniqueness, local and global uniqueness, and unitary uniqueness. In the homogeneous condition, all stimuli as targets and distractors consisted of the same global letter composed of the same local letter, e.g., a large letter of “A” that composed of small letter of “A.” The same one compound stimulus where the global letter and local letter were congruent in each trial was chosen randomly from the eight congruent large letters (A, E, F, H, L, S, T, and V). The homogeneous condition provided the performance baseline for comparison. In the local-uniqueness condition, all the eight stimuli were the same global letter, which consisted of different local letters, respectively. That is, the global properties of the stimuli were the same but their local properties were distinct. In the global-uniqueness condition, the eight stimuli were different global letters consisted of the same local letters. That is, the stimuli were the same at the local level but different at the global level. In the local-and-global uniqueness condition, the eight stimuli were different global letters which was composed of different local letters, respectively. That is, the objects’ global and local properties were both distinct. In the unitary-uniqueness condition, the eight objects were different unitary letters. Table 1 presents the details of the experiment design. The dependent variable was tracking accuracy, defined as the average proportion of correctly identified targets.

**Table 1:**
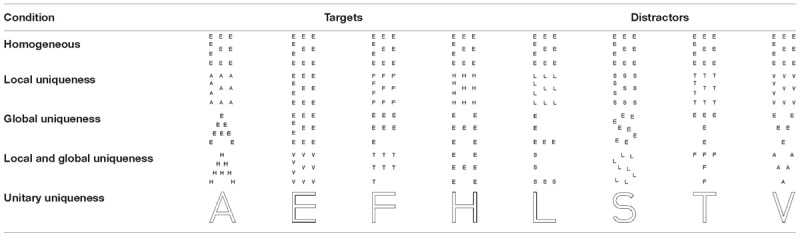
The five conditions of Experiment 1.

### Procedures

**FIGURE 1:**
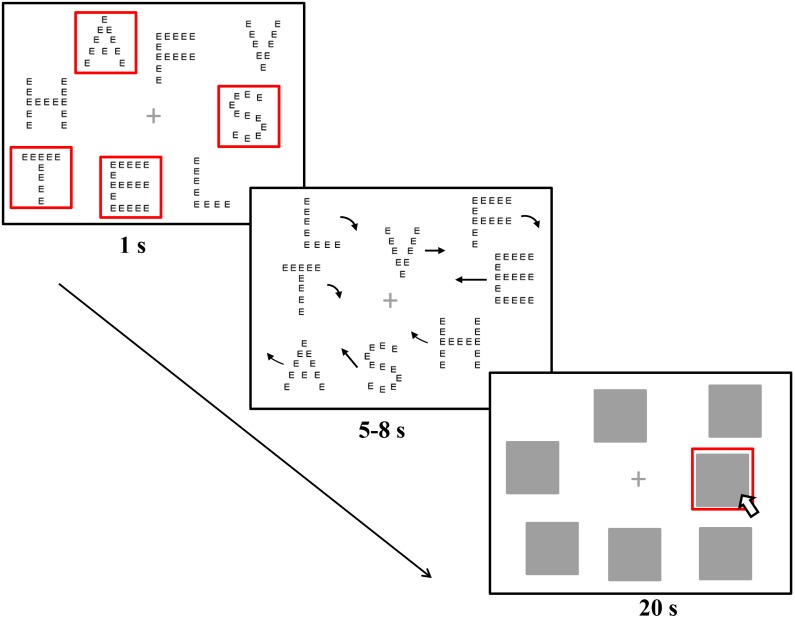
Sample illustrations of a trial in the global-uniqueness condition of Experiment 1. The compound stimuli surrounded by red squares were the targets, indicated by flashing five times in 1 s during the experiment.

Observers sat about 55–60 cm away from the monitors while the chair was kept in a fixed position. Observers were required to reduce their head motion and to keep still during the whole experiment. Although a chin rest was not used, at this viewing distance, each pixel could be seen as subtending approximately 0.04 degrees of the visual angle. At the start of each trial, a gray fixation cross and eight pictures were displayed. No special instructions were given concerning fixation. Four of eight pictures, as the targets, flashed five times in 1 s, when the observers could identify and distinguish them from the distractors. Then all of the pictures began to move randomly and unpredictably, and the movement stopped at a random point within 5–8 s. At the same time of motion ending, the pictures were masked by gray squares subtending 90 pixels × 90 pixels (3.6° × 3.6°). The observers selected all four targets with the mouse within 20 s. The selected objects were surrounded by red frames. Observers pressed the space bar to continue to the next trial (see the sample trial procedure in Figure 1). The experiment consisted of totally 100 trials (20 trials × 5 conditions) that presented randomly.

### Results and Discussion

**FIGURE 2:**
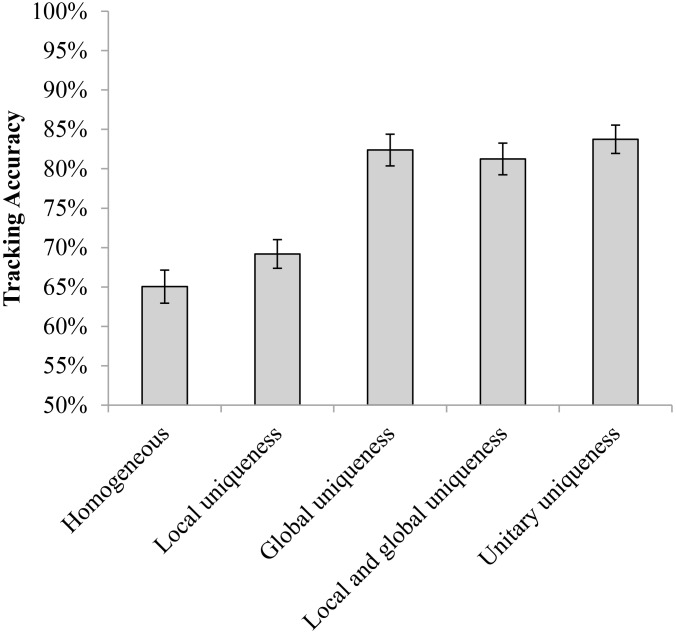
The tracking accuracies of the five conditions in Experiment 1 (error bars show ±1 standard error of the mean).

Figure 2 presents the results of Experiment 1. A repeated measures analysis of variance (ANOVA) indicated a significant main effect [*F*(4,64) = 37.070, *p* < 0.001, $\eta_{p}^{2}$ = 0.699, Power (1–β) > 99%]. *Post hoc* tests with Bonferroni correction revealed that the tracking accuracy of observers in the local-uniqueness condition was significantly higher than the accuracy in the homogeneous condition [local-uniqueness vs. homogeneous: *t*(16) = 3.415, *p* = 0.035 (Bonferroni adjusted *p*-value, the same below), Cohen’s *d* = 0.53], providing evidence that the distinctiveness of the objects’ local properties could facilitate observers’ tracking when their global properties were identical. There was no significant difference among the global-uniqueness, local-and-global uniqueness, and unitary-uniqueness conditions with pairwise comparison (all *p* = 1.000). Furthermore, the tracking accuracies in these three conditions were all significantly higher than those in the homogeneous condition and the local-uniqueness condition [global-uniqueness vs. homogeneous: *t*(16) = 6.207, *p* < 0.001, Cohen’s *d* = 2.11; local-and-global uniqueness vs. homogeneous: *t*(16) = 8.105, *p* < 0.001, Cohen’s *d* = 1.98; unitary-uniqueness vs. homogeneous: *t*(16) = 7.676, *p* < 0.001, Cohen’s *d* = 2.40; global-uniqueness vs. local-uniqueness: *t*(16) = 5.267, *p* = 0.001, Cohen’s *d* = 1.72; local-and-global uniqueness vs. local-uniqueness: *t*(16) = 6.847, *p* < 0.001, Cohen’s *d* = 1.57; unitary-uniqueness vs. local-uniqueness: *t*(16) = 6.543, *p* < 0.001, Cohen’s *d* = 2.01]. These results suggest that the distinctiveness of global properties of compound stimuli could be recognized by observers during motion and facilitated the tracking performance to the accuracy level as in the unitary-uniqueness condition. Since observers’ tracking performance in the local-and-global uniqueness condition did not improve upon the global-uniqueness condition, we inferred that observers mainly used the uniqueness of global properties to aid tracking.

## Experiment 2

In Experiment 2, we used the sparse compound stimuli composed of fewer local elements to answer the same questions as in Experiment 1. Previous research has revealed that stimulus sparsity can determine the relative ease of processing global and local properties of compound stimuli. The global identities of compound stimuli with few local elements may be more difficult for observers to extract than those with many local elements (Martin, 1979; Navon, 1983; Kimchi, 1992). We tested that when the global identities were difficult to retrieve and recognize, whether the uniqueness of global properties or that of local properties could facilitate object-based tracking.

### Methods

#### Participants

Twenty-two students (16 female, 6 male), 18–28 years old (mean age = 21.14, *SD* = 2.68) with normal or corrected-to-normal vision, participated in the experiment. None of them had participated in Experiment 1. Other particulars were the same as in Experiment 1.

#### Design, Equipment, Stimuli and Procedures

The design, equipment and procedures were the same as those of Experiment 1, except that the sparse compound stimuli patterns with fewer local elements were used (see Tables 2, 3 for more details). The total number of local elements was decreased from 90 in Experiment 1 to 55 in Experiment 2. Furthermore, the stimuli’s moving speed decreased from 17°/s in Experiment to about 14.5°/s in Experiment 2.

**Table 2:** The number of the local elements in Experiments 1 and 2.

	Experiment 1	Experiment 2
A	10	8
E	17	10
F	13	7
H	13	7
L	8	5
S	11	8
T	9	5
V	9	5

**Table 3:**
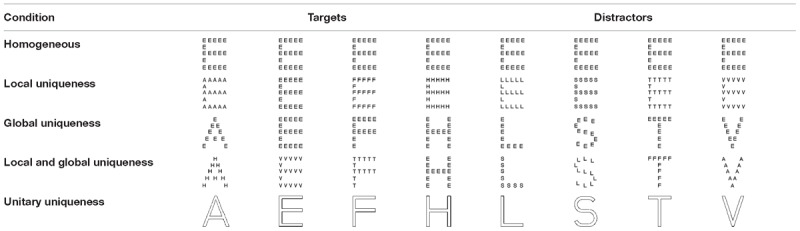
The five conditions of Experiment 2.

### Results and Discussion

**FIGURE 3:**
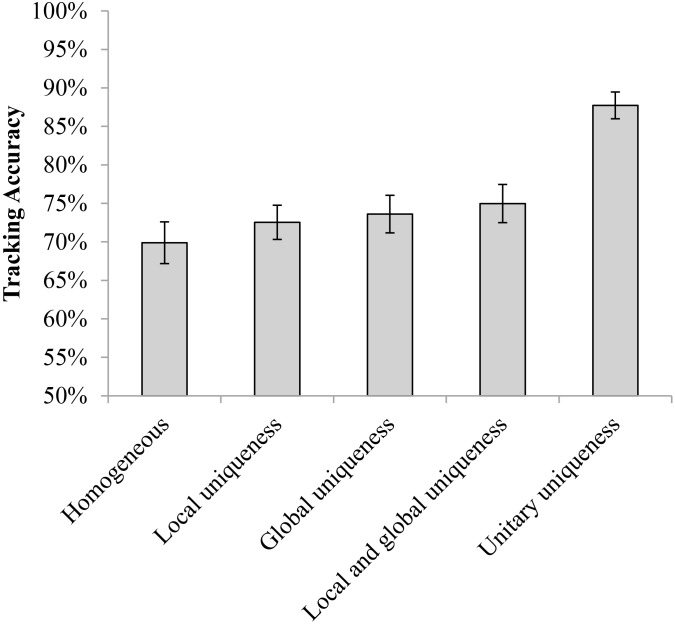
The tracking accuracies of the five conditions in Experiment 2 (error bars show ±1 standard error of the mean).

Figure 3 shows the results from Experiment 2. A repeated measures ANOVA indicated a significant main effect [*F*(4, 84) = 43.729, *p* < 0.001, $\eta_{p}^{2}$ = 0.676, Power (1–β) > 99%]. *Post hoc* tests with Bonferroni correction showed that the tracking accuracy of observers in the unitary-uniqueness condition was significantly higher than the accuracies in all the other four conditions [unitary-uniqueness vs. homogeneous: *t*(21) = 10.328, *p* < 0.001 (Bonferroni adjusted *p*-value, the same below), Cohen’s *d* = 1.71; unitary-uniqueness vs. local-uniqueness: *t*(21) = 8.930, *p* < 0.001, Cohen’s *d* = 1.66; unitary-uniqueness vs. global-uniqueness: *t*(21) = 8.262, *p* = 0.001, Cohen’s *d* = 1.45; unitary-uniqueness vs. local-and-global uniqueness: *t*(21) = 7.496, *p* = 0.001, Cohen’s *d* = 1.30]. The tracking accuracy in the local-and-global uniqueness condition was significantly higher than that in the homogeneous condition [*t*(21) = 3.884, *p* = 0.009, Cohen’s *d* = 0.43]. One exception is that, pairwise comparisons between the homogeneous, local-uniqueness, global-uniqueness and local-and-global uniqueness conditions showed no significant difference (*p*s > 0.05). These results suggest that when both the local and global properties of the sparse compound stimuli were distinct from each other, observers’ tracking performance showed a significant improvement compared to the homogeneous condition (5.09% of increment in tracking accuracy). We did not find a significant facilitation effect of the unique local properties or unique global properties under the current experiment situations. However, since the tracking loads of the present MOT task were at the moderate level (the tracking accuracies were from 69 to 75% in the four compound stimuli conditions), the results might be different when the tracking loads were lower or higher. Therefore, it would be helpful to test this issue under different tracking loads or different task difficulties in the future. Moreover, results of Experiment 2 showed different conclusions from that of Experiment 1. The detailed reasons of these discrepant results will be further discussed in the general discussion section.

## General Discussion and Conclusion

The present study used compound stimuli and tested whether the global or local properties played the main role in the uniqueness-facilitation effect in the MOT task. In light of the results from Experiment 1, we infer that the distinctiveness of local properties can facilitate tracking when the global identities of the compound stimuli are identical, even the facilitation is small (4.15% of increment in tracking accuracy relative to the homogeneous condition). However, when the stimuli’s global properties are distinct from each other—whether the local properties being unique or not—observers’ tracking performance can be as good as that in the unitary-uniqueness condition. Results from Experiment 2 show that in the sparse compound stimuli situations, a uniqueness-facilitation effect was identified under the condition when both the local and global properties were distinct from each other. However, the unique local properties or unique global properties alone did not show any significant facilitation effect on observers’ tracking performance in the current experiment. The present study revealed that both the unique local properties and the unique global properties could facilitate tracking when the compound stimuli consisted of many local elements, but the global properties played the main role in the uniqueness-facilitation effect.

### The Effect of Unique Local Properties on Tracking

The present study demonstrated that when the compound stimuli with many local elements had identical global properties, their distinct local properties could slightly facilitate tracking. Specifically, when the global properties of the compound stimuli were undistinguishable, the unique local properties could be used to aid tracking. This finding suggests that in the dynamic scenes, the uniqueness of the bottom level in the hierarchical network (i.e., local properties) could be captured and utilized during tracking. However, this uniqueness-facilitation effect of the local properties seemed to be apparent only when the global properties were identical. When the global properties of the compound stimuli were distinct, observers mainly used the unique global identities to aid tracking. This claim comes from two pieces of evidence: one is that observers’ tracking performance in the global-uniqueness condition was significantly better than that in the local-uniqueness condition. The other is that the tracking accuracies in the local-and-global uniqueness condition and the global-uniqueness had no significant difference. Our results also suggest that the uniqueness-facilitation effect of the local properties seems not to be additive, since the tracking accuracy in the global-uniqueness condition (82.38%) was close to the tracking accuracy in the local-and-global uniqueness condition (81.25%). Furthermore, the lack of additivity cannot be ascribed to floor/ceiling effects because observers’ tracking performance in Experiment 1 ranged from 64 to 84%.

### The Global Superiority Effect in the MOT Task

As mentioned above, when the global properties of the compound stimuli were distinct from each other, observers mainly relied on the unique global identities to aid tracking (Results of Experiment 1). These results reveal a global superiority effect in the MOT task. That is, the uniqueness of the global properties or the uniqueness of the configurations plays a major role in facilitating tracking.

One might argue that the global superiority effect only exists because the size of the global configuration was much larger than that of the local elements, which is a fact of nature since the global properties are properties of a higher-level unit in the hierarchical patterns (Navon, 2003; Wagemans et al., 2012b). However, the global configurations of the sparse compound stimuli in Experiment 2 had the same sizes as that of the compound stimuli in Experiment 1, but no global superiority effect was found in the global-uniqueness condition. Therefore, this alternative explanation of relative size of global and local properties could be ruled out to some extent. We conjecture that the global superiority effect is based on the same mechanism of how unique features or identities facilitate tracking (Makovski and Jiang, 2009a,b; Liu et al., 2012). This mechanism might consist of two stages: in the first stage, observers perceived the compound stimuli as the meaningful objects and retrieved their global identities. In the second stage, these global identities could be stored in visual working memory. When one or more targets were lost during tracking, observers could use the stored identities to find them back (Horowitz et al., 2007; Makovski and Jiang, 2009a,b). But of course, this hypothesis will need to be verified in future studies.

One issue we noticed is that results of Experiment 1 revealed a global superiority effect, while no significant facilitation effect of unique global properties on tracking was found in Experiment 2. The major difference between Experiments 1 and 2 is the adopted compound stimuli. Specifically, the compound stimuli in Experiment 1 consisted of more local elements while the compound stimuli in Experiment 2 consisted of fewer local elements. Thus, the number of local elements might exert an influence on the global superiority effect. Although lacking sufficient empirical evidence from the current study, we speculate that when the compound stimuli has many local elements, the contour of the global letters is better, making it easy for the observers to extract their identities and aid tracking. On the contrary, when the compound stimuli composes of few local elements, the contours of their global structures lack tight connection, which might constrain the observers from recognizing their global forms and retrieving their identities (Martin, 1979; Navon, 1983, 2003). This speculation could also be further tested in our next study.

The global superiority effect in the MOT task suggests that: on one hand, observers may mainly rely on visual objects’ global properties to retrieve and recognize their identities in dynamic scenes; on the other hand, the grouping principles of proximity and good continuation for contour integration (Wagemans et al., 2012a) may also be applied in the motion scenes. While the visual elements are with proximity and good continuation (e.g., the compound stimuli with many local elements in Experiment 1), observers may be able to integrate their contours and retrieve their global identities even when the visual patterns are in a continuous and random motion.

Although the present study used compound stimuli similar to Navon’s (1977), the goal of the present study was not to explore the orders of processing of the global and local properties, or their interference with each other. Therefore, we will not discuss more about the processing mechanism of compound stimuli. Compared with real-world stimuli, the compound stimuli provide an elegant way to manipulate the distinctiveness of the local and global properties, respectively, except that, the other factors could be manipulated to be comparable across stimuli. However, the lack of external validity with the compound stimuli and the corresponding conclusions need to be further explored in future studies.

## Ethics Statement

This study was carried out in accordance with the recommendations of ‘the institutional review board (ethics committee) of the Faculty of Psychology at Beijing Normal University’ with written informed consent from all subjects. All subjects gave written informed consent in accordance with the Declaration of Helsinki. The protocol was approved by the institutional review board (ethics committee) of the Faculty of Psychology at Beijing Normal University.

## Author Contributions

LW and XZ conceived and designed the experiments. LW performed the experiments and analyzed the data. LW, XZ, ZL, BH, and XL contributed to the writing of the manuscript.

## Conflict of Interest Statement

The authors declare that the research was conducted in the absence of any commercial or financial relationships that could be construed as a potential conflict of interest.

## References

[B1] Cohen M. A. Pinto Y. Howe P. D. L. Horowitz T. S. (2011). The what–where tradeoff in multiple identity tracking. *Attent. Percept. Psychophys.* 73 1422–1434. 10.3758/s13414-011-0089-7 21380611

[B2] Erlikhman G. Keane B. Mettler E. Horowitz T. Kellman P. (2013). Automatic feature-based grouping during multilple object tracking. *J. Exp. Psychol. Hum. Percept. Perform.* 39 1625–1637. 10.1037/a0031750 23458095 PMC3901520

[B3] Faul F. Erdfelder E. Lang A.-G. Buchner A. (2007). G^∗^Power 3: a flexible statistical power analysis program for the social, behavioral, and biomedical sciences. *Behav. Res. Methods* 39 175–191. 10.3389/fpsyg.2012.00307 17695343

[B4] Horowitz T. S. Klieger S. B. Fencsik D. E. Yang K. K. Alvarez G. A. Wolfe J. M. (2007). Tracking unique objects. *Percept. Psychophys.* 69 172–184. 10.3758/bf03193740 17557588

[B5] Howe P. Holcombe A. (2012). The effect of visual distinctiveness on multiple object tracking performance. *Front. Percept. Sci.* 3:307. 10.3389/fpsyg.2012.00307 22969738 PMC3427910

[B6] Kimchi R. (1992). The primacy of wholistic processing and the global/local paradigm: a critical review. *Psychol. Bull.* 112 24–38. 10.1037//0033-2909.112.1.24 1529037

[B7] Liu T. W. Chen W. F. Liu C. H. Fu X. L. (2012). Benefits and costs of uniqueness in multiple object tracking: the role of object complexity. *Vis. Res.* 66 31–38. 10.1016/j.visres.2012.06.009 22750543

[B8] Makovski T. Jiang Y. V. (2009a). Feature binding in attentive tracking of distinct objects. *Vis. Cogn.* 17 180–194. 10.1080/13506280802211334 19492017 PMC2689094

[B9] Makovski T. Jiang Y. V. (2009b). The role of visual working memory in attentive tracking of unique objects. *J. Exp. Psychol. Hum. Percept. Perform.* 35 1687–1697. 10.1037/a0016453 19968429 PMC2792568

[B10] Martin M. (1979). Local and global processing: the role of sparsity. *Mem. Cogn.* 7 476–484. 10.3758/bf03198264

[B11] Navon D. (1977). Forest before trees: the precedence of global features in visual perception. *Cogn. Psychol.* 9 353–383. 10.1016/0010-0285(77)90012-3

[B12] Navon D. (1983). How many trees does it take to make a forest? *Perception* 12 239–254. 10.1068/p120239 6669451

[B13] Navon D. (2003). What does a compound letter tell the psychologist’s mind? *Acta Psychol.* 114 273–309. 10.1016/j.actpsy.2003.06.002 14670701

[B14] Pylyshyn Z. W. (2004). Some puzzling findings in multiple object tracking: I. Tracking without keeping track of object identities. *Vis. Cogn.* 11 801–822. 10.1080/13506280344000518

[B15] Pylyshyn Z. W. (2006). Some puzzling findings in multiple object tracking: II. Inhibition of moving nontargets. *Vis. Cogn.* 14 175–198. 10.1080/13506280544000200

[B16] Pylyshyn Z. W. Storm R. W. (1988). Tracking multiple independent targets: evidence for a parallel tracking mechanism. *Spat. Vis.* 3 179–197. 10.1163/156856888x00122 3153671

[B17] Ren D. Chen W. Liu C. H. Fu X. (2009). Identity processing in multiple-face tracking. *J. Vis.* 9 1–15. 10.1167/9.5.18 19757896

[B18] Scholl B. J. Pylyshyn Z. W. (1999). Tracking multiple items through occlusion: clues to visual objecthood. *Cogn. Psychol.* 38 259–290. 10.1006/cogp.1998.0698 10090804

[B19] Scholl B. J. Pylyshyn Z. W. Feldman J. (2001). What is a visual object? Evidence from target merging in multiple-object tracking. *Cognition* 80 159–177. 10.1016/s0010-0277(00)00157-8 11245843

[B20] vanMarle K. Scholl B. J. (2003). Attentive tracking of objects vs. substances. *Psychol. Sci.* 14 498–504. 10.1111/1467-9280.03451 12930483

[B21] Wagemans J. Elder J. H. Kubovy M. Palmer S. E. Peterson M. A. Singh M. (2012a). A century of Gestalt psychology in visual perception: I. Perceptual grouping and figure–ground organization. *Psychol. Bull.* 138 1172–1217. 10.1037/a0029333 22845751 PMC3482144

[B22] Wagemans J. Feldman J. Gepshtein S. Kimchi R. Pomerantz J. R. van der Helm PA (2012b). A century of gestalt psychology in visual perception: ii. conceptual and theoretical foundations. *Psychol. Bull.* 138 1218–1252. 10.1037/a0029334 22845750 PMC3728284

[B23] Wang C. Zhang X. Li Y. Lyu C. (2016). Additivity of feature-based and symmetry-based grouping effects in multiple object tracking. *Front. Psychol.* 7:657. 10.3389/fpsyg.2016.00657 27199875 PMC4854980

[B24] Wei L. Zhang X. Li Z. Liu J. (2018). The semantic category-based grouping in the multiple identity tracking task. *Attent. Percept. Psychophys.* 80 118–133. 10.3758/s13414-017-1420-8 28956323

[B25] Wei L. Zhang X. Lyu C. Hu S. Li Z. (2017). Brain activation of semantic category-based grouping in multiple identity tracking task. *PLoS One* 12:e0177709. 10.1371/journal.pone.0177709 28505166 PMC5432174

[B26] Wei L. Zhang X. Lyu C. Li Z. (2016). The categorical distinction between targets and distractors facilitates tracking in multiple identity tracking task. *Front. Psychol.* 7:589. 10.3389/fpsyg.2016.00589 27199824 PMC4849363

